# Molecular analysis of 76 Chinese hemophilia B pedigrees and the identification of 10 novel mutations

**DOI:** 10.1002/mgg3.1482

**Published:** 2020-09-01

**Authors:** Limin Huang, Liyan Li, Sheng Lin, Juanjuan Chen, Kun Li, Dongmei Fan, Wangjie Jin, Yihong Li, Xu Yang, Yufeng Xiong, Fenxia Li, Xuexi Yang, Ming Li, Qiang Li

**Affiliations:** ^1^ Institute of Antibody Engineering School of Laboratory Medicine and Biotechnology Southern Medical University Guangzhou China; ^2^ Technology Center of Prenatal Diagnosis and Genetic Diseases Diagnosis, Department of Gynecology and Obstetrics Nanfang Hospital Southern Medical University Guangzhou China; ^3^ Laboratory of Molecular Medicine Shenzhen Health Development Research Center Shenzhen China; ^4^ Clinical Innovation & Research Center (CIRC) Shenzhen Hospital of Southern Medical University Shenzhen China; ^5^ Department of Clinical Laboratory Guangdong Women and Children Hospital Guangzhou China; ^6^ The Department of Laboratory Medicine Nanfang Hospital Southern Medical University Guangzhou China

**Keywords:** *F9*, hemophilia B, molecular diagnosis, next‐generation sequencing

## Abstract

**Background:**

Hemophilia B (HB) is an X‐linked recessive inherited bleeding disorder caused by mutations in the *F9* gene that lead to plasma factor IX deficiency. To identify the causative mutations in HB, a molecular analysis of HB pedigrees in China was performed.

**Methods:**

Using next‐generation sequencing (NGS) and an in‐house bioinformatics pipeline, 76 unrelated HB pedigrees were analyzed. The mutations identified were validated by comparison with the results of Sanger sequencing or Multiplex Ligation‐dependent Probe Amplification assays. The pathogenicity of the causative mutations was classified following the American College of Medical Genetics and Genomics guidelines.

**Results:**

The mutation detection rate was 94.74% (72/76) using NGS. Of the 76 HB pedigrees analyzed, 59 causative variants were found in 72 pedigrees, with 38 (64.41%) missense mutations, 9 (15.25%) nonsense mutations, 2 (3.39%) splicing mutations, 5 (8.47%) small deletions, 4 (6.78%) large deletions, and 1 intronic mutation (1.69%). Of the 59 different *F9* mutations, 10 were novel: c.190T>G, c.199G>T, c.290G>C, c.322T>A, c.350_351insACAATAATTCCTA, c.391+5delG, c.416G>T, c.618_627delAGCTGAAACC, c.863delA, and c.1024_1027delACGA. Of these 10 novel mutations, a mosaic mutation, c.199G>T(p.Glu67Ter), was identified in a sporadic HB pedigree. Using *in‐silico* analysis, these novel variants were predicted to be disease‐causing. However, no potentially causative mutations were found in the *F9* coding sequences of the four remaining HB pedigrees. In addition, two HB pedigrees carrying additional *F8*/*F9* mutations were discovered.

**Conclusion:**

The identification of these mutations enriches the spectrum of *F9* mutations and provides further insights into the pathogenesis of HB in the Chinese population.

## Graphical abstract

Huang et al analyzed the spectrum of F9 gene in 76 unrelated HB Chinese pedigrees using NGS. Fifty‐nine causative variants were found, with 64.41% missense mutations, 15.25% nonsense mutations, 3.39% splicing mutations, 8.47% small deletions, 6.78% large deletions and 1.69% intronic mutation. Of these 59 F9 variants, 10 were novel.

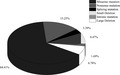

## 1 INTRODUCTION

Hemophilia B (HB; OMIM 306900) is a rare X‐linked recessive hemorrhagic disorder caused by a deficiency or abnormality in coagulation factor IX (FIX) due to mutations in the *F9* gene, affecting 1 in 30,000 male live births (Bolton‐Maggs & Pasi, 2003). HB is diagnosed based on clinical manifestations and laboratory results. Based on residual plasma FIX activity, HB is classified as severe (FIX:C <1%), moderate (FIX:C 1%–5%), or mild (FIX:C 5%–40%) (Goodeve, 2015). At present, under clinical or preclinical investigation, the only radical cure for HB, gene therapy, is not yet mature (Hasbrouck & High, 2008; Maitituoheti et al., 2011). And the main treatment is FIX replacement therapy (Srivastava et al., 2013), which places a heavy burden on the family and society. The detection of causative genetic variants in HB families has attracted the attention of clinicians to predict the development of inhibitors and provide genetic counseling.

As of February 2020, more than 1244 *F9* mutations have been recorded in the FIX Variant Database (http://www.factorix.org/). These mutations include point mutations, deletions, insertions, duplications, insertions, and deletions(InDel), complex mutations, and polymorphisms. Most of the reported mutations are point mutations, which account for more than 70% of these mutations (McVey et al., 2020).

Based on the reported spectrum of *F9* mutations in different populations—for example, Americans, Colombians, Italians, Canadians, etc.—the spectrum of *F9* mutations differs across populations (Belvini et al., 2005; Chen et al., 2020; Li et al., 2014; Natalia, Jayne, Shawn, Paula, & David, 2013; Parrado Jara, Yunis Hazbun, Linares, & Yunis Londoño, 2020). Therefore, enriching the FIX variant database enables us to analyze the distribution of *F9* mutations in different populations and provides more detailed annotation information on these variants. Here, we performed genetic analyses of 76 unrelated HB pedigrees in China and examined the *F9* gene to enrich the spectrum of *F9* mutations in China.

## 2 MATERIALS AND METHODS

### 2.1 Ethical statement

Informed consent was obtained from all study participants. This study was approved by the Ethics Committee of Nanfang Hospital (Approval no. NFEC‐2016‐035) and in accordance with the Principles of the Declaration of Helsinki.

### 2.2 Subjects

The study analyzed 285 subjects from 76 unrelated Chinese pedigrees with HB diagnosed at Nanfang Hospital, Southern Medical University (Guangzhou, China) from March 2017 to December 2019. Basic information of this cohort is described in Table S1.

### 2.3 Sample collection and DNA extraction

Peripheral blood was collected from each participant in EDTA tubes. Genomic DNA was extracted from 200 μl of peripheral blood using Nucleic Acid Isolation or Purification reagent (Guangzhou Darui Biotechnology). DNA concentrations were assessed using the Qubit dsDNA HS Assay Kit in a Qubit 3.0 Fluorometer (Life Technologies) following the manufacturer's instructions, and the extracted DNA was stored at –20℃.

### 2.4 Library preparation and next‐generation sequencing

Using a designed panel targeting the most important regions of the *F8*, *F9*, and *VWF* genes (coding sequences, untranslated regions, and 10 bp of exon‐intron junction regions, GenBank accession no. NM_000132.3, NM_000133.2, NM_000552.3, respectively, human genome hg19), libraries were prepared using an Ion AmpliSeq Library Kit 2.0 (Life Technologies) following the manufacturer's instructions. In this process, each library was labeled with a unique barcode using Ion Xpress Barcode Adaptors 1‐96 Kit (Life Technologies) and diluted to a concentration of ~100 pM. Subsequently, libraries to be sequenced were pooled in equimolar proportions and subjected to emulsion PCR on an Ion OneTouch™ 2 Instrument (Life Technologies). After template enrichment, the positive templates were loaded on a semiconductor chip and sequenced by synthesizing on an Ion Torrent sequencing platform (Life Technologies).

### 2.5 Next‐generation sequencing data analysis

The raw sequencing data were processed using Ion Torrent Suite v5.4.0 (Life Technologies). After running the Variant Caller plug‐in, a VCF file for each sample was generated. For variant annotation and interpretation, an in‐house bioinformatics pipeline that integrates several population and mutation databases (dbnsfp33a, 1000Genomes, ExAC, gnomAD, ClinVar, and HGMD) and bioinformatics tools was run. The clinical significance of each identified variant was characterized according to the American College of Medical Genetics and Genomics (ACMG) criteria as follows: pathogenic, likely pathogenic, variant of unknown significance, likely benign, or benign. Candidate variants were checked in the EAHAD *F9* variant database (http://f9‐db.eahad.org), LOVD (https://databases.lovd.nl/shared/genes/F9), and CHBMP (http://www.cdc.gov/hemophiliamutations/). SIFT (Kumar, Henikoff, & Ng, 2009), PolyPhen‐2 (Adzhubei et al., 2010), and PROVEAN (Choi, Sims, Murphy, Miller, & Chan, 2012) were used to evaluate the deleterious nature of the novel missense mutations. All causative mutations in each pedigree identified by next‐generation sequencing (NGS) were validated by comparison with the results of Sanger sequencing or Multiplex Ligation‐dependent Probe Amplification assays.

An Ion Reporter workflow was created to call the copy number variation (CNV) using Ion Reporter Software version 5.10 (Thermo Fisher Scientific). CNV detection algorithm of Ion Reporter is based on the Hidden Markov Model (HMM), the algorithm uses read coverage to detect the copy number states. The coverage is corrected for GC bias and contrasted against a “baseline” coverage that is constructed from one or more controls. Subsequently, copy number segments and their ploidies were computed.

All variants identified were further checked manually using the Integrative Genomics Viewer (IGV, Broad Institute) with BAM files.

## 3 RESULTS

### 3.1 Mutation spectrum

**FIGURE 1:**
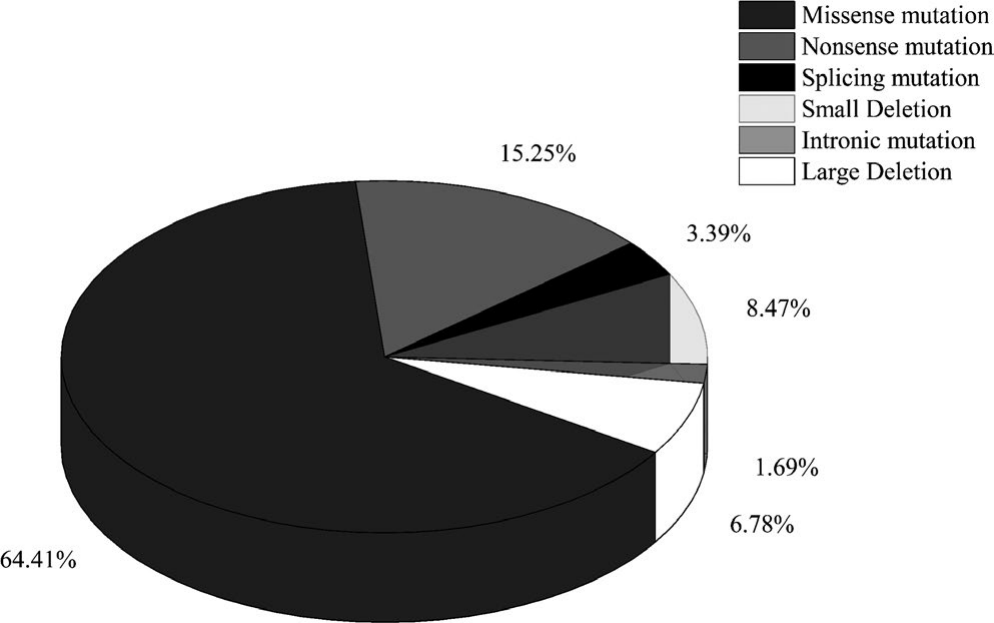
The frequency of the 59 variants identified in 76 Chinese hemophilia B (HB) pedigrees: 38 (64.41%) were missense mutation, nine (15.25%) were nonsense mutations, five were small deletions (8.47%), four were large deletions (6.78%), and two were splicing mutations (3.39%). The remaining one was an intronic mutation

On average, 98.79% of the target region had at least 20× coverage required for confident variant calls and 97.37% of the target region had more than 100× coverage. From 285 cases representing 76 HB pedigrees, 59 different variants were identified in 72 pedigrees: 38 (64.41%) missense mutations, nine (15.25%) nonsense mutations, five small deletions (8.47%), four large deletions (6.78%; Figure 1), and two splicing mutations (3.39%). In addition, an intronic mutation classified as variants of unknown significance was detected in two carriers and one patient from HB423. The details of these mutations are listed in Table 1. Of the 59 identified variants, 49 had been reported and 10 were novel mutations. No mutations in the targeted *F9* region were found in four pedigrees.

**TABLE 1:** Detailed description of the mutations identified in 76 hemophilia B (HB) Chinese pedigrees

HGVS cDNA Name	HGVS Protein Name	Mutation Type	Exon	Domain	CpG	Significance*	Previously reported	Family Number
c.26C>G	p.Ala9Gly	Missense	Exon1	Signal Peptide	–	3	Yes	1
c.59T>C	p.Leu20Ser	Missense	Exon1	Signal Peptide	N	3	Yes	1
c.88+1G>T	–	Splicing	Intron 1	–	N	5	Yes	1
c.127C>T	p.Arg43Trp	Missense	Exon2	Pro‐Peptide	Y	4	Yes	6
c.128delG	p.Arg43fs	Deletion	Exon2	Pro‐Peptide	Y	5	Yes	1
c.128G>A	p. Arg43Gln	Missense	Exon2	Pro‐Peptide	Y	4	Yes	3
c.155T>C	p.Leu52Ser	Missense	Exon2	Gla	N	4	Yes	1
c.173G>C	p.Gly58Ala	Missense	Exon2	Gla	N	4	Yes	1
c.188A>G	p.Glu63Gly	Missense	Exon2	Gla	N	4	Yes	1
c.190T>G	p.Cys64Gly	Missense	Exon2	Gla	–	4	No	1
c.199G>T	p. Glu67Ter	Nonsense	Exon2	Gla	N	5	No	1
c.206G>A	p.Cys69Tyr	Missense	Exon2	Gla	N	4	Yes	1
c.223C>T	p.Arg75Ter	Nonsense	Exon2	Gla	Y	5	Yes	1
c.290G>C	p.Cys97Ser	Missense	Exon4	EGF1	N	4	No	1
c.304T>C	p.Cys102Arg	Missense	Exon4	EGF1	N	4	Yes	1
c.322T>A	p.Cys108Ser	Missense	Exon4	EGF1	N	4	No	1
c.323G>A	p.Cys108Tyr	Missense	Exon4	EGF1	N	4	Yes	1
c.340T>C	p.Ser114Pro	Missense	Exon4	EGF1	N	4	Yes	1
c.344A>C	p.Tyr115Ser	Missense	Exon4	EGF1	N	4	Yes	1
c.350_351insACAATAATTCCTA	p.Cys117_Trp118delinsTer	Nonsense	Exon4	EGF1	N	5	No	1
c.383G>C	p.Cys128Ser	Missense	Exon4	EGF1	N	4	Yes	1
c.391+2T>C	–	Splicing	Intron 4	–	N	5	Yes	1
c.391+5delG	–	Intronic	Intron 4	–	N	3	No	1
c.416G>T	p.Gly139Val	Missense	Exon5	EGF2	N	4	No	1
c.464G>T	p.Cys155Phe	Missense	Exon5	EGF2	N	4	Yes	1
c.484C>T	p.Arg162Ter	Nonsense	Exon5	EGF2	Y	5	Yes	1
c.523C>T	p.Pro175Ser	Missense	Exon6	EGF2	–	4	Yes	1
c.618_627delGGTTTCAGCT	p.Glu206fs	Deletion	Exon6	Act‐Peptide	N	5	No	2
c.676C>T	p.Arg226Trp	Missense	Exon6	Act‐Peptide	Y	4	Yes	3
c.677G>A	p.Arg226Gln	Missense	Exon6	Act‐Peptide	Y	4	Yes	1
c.677G>T	p.Arg226Leu	Missense	Exon6	Act‐Peptide	Y	4	Yes	1
c.682G>T	p.Val228Phe	Missense	Exon6	Serine Protease	N	4	Yes	1
c.716C>T	p.Phe239Leu	Missense	Exon6	Serine Protease	–	4	Yes	1
c.719G>A	p.Trp240Ter	Nonsense	Exon6	Serine Protease	N	5	Yes	1
c.767T>C	p.Ile256Thr	Missense	Exon7	Serine Protease	N	4	Yes	1
c.781T>C	p.Trp261Arg	Missense	Exon7	Serine Protease	N	4	Yes	1
c.782G>T	p.Trp261Leu	Missense	Exon7	Serine Protease	N	4	Yes	1
c.863delA	p.Glu288fs	Deletion	Exon8	Serine Protease	–	5	No	1
c.892C>T	p.Arg298Ter	Nonsense	Exon8	Serine Protease	Y	5	Yes	1
c.1024_1027delTCGT	p.Thr342fs	Deletion	Exon8	Serine Protease	N	5	No	1
c.1069G>A	p.Gly357Arg	Missense	Exon8	Serine Protease	N	4	Yes	1
c.1095delA	p.Ser365fs	Deletion	Exon8	Serine Protease	–	5	Yes	1
c.1132G>T	p.Asp378Tyr	Missense	Exon8	Serine Protease	N	4	Yes	2
c.1135C>T	p.Arg379Ter	Nonsense	Exon8	Serine Protease	Y	5	Yes	3
c.1150C>T	p.Arg384Ter	Nonsense	Exon8	Serine Protease	Y	5	Yes	1
c.1169T>A	p.Ile390Asn	Missense	Exon8	Serine Protease	N	4	Yes	1
c.1225G>A	p.Gly409Arg	Missense	Exon8	Serine Protease	N	4	Yes	1
c.1231A>G	p.Ser411Gly	Missense	Exon8	Serine Protease	N	4	Yes	1
c.1237G>A	p.Gly413Arg	Missense	Exon8	Serine Protease	N	4	Yes	1
c.1238G>A	p.Gly413Glu	Missense	Exon8	Serine Protease	N	4	Yes	1
c.1277C>G	p.Thr426Ser	Missense	Exon8	Serine Protease	N	4	Yes	1
c.1294G>A	p.Gly432Ser	Missense	Exon8	Serine Protease	N	4	Yes	2
c.1295G>T	p.Gly432Val	Missense	Exon8	Serine Protease	N	4	Yes	1
c.1305T>A	p.Cys435Ter	Nonsense	Exon8	Serine Protease	N	5	Yes	1
c.1307C>T	p.Ala436Val	Missense	Exon8	Serine Protease	N	4	Yes	1
Del. exons2‐4	–	Large Deletion	Exon2‐4	Gla‐EGF1	–	–	Yes	1
Del. exon6	–	Large Deletion	Exon6	Act‐Peptide	–	–	Yes	1
Del. exons1‐5	–	Large Deletion	Exon1‐5	Signal Peptide ‐ EGF2	–	–	Yes	1
Del. exon4	–	Large Deletion	Exon4	EGF1	–	–	Yes	1

* In the “significant” column, 5 refers to “pathogenic,” 4 refers to “likely pathogenic,” 3 refers to “variant of unknown significance.”

### 3.2 Novel mutations

Of the 59 variants identified in 76 HB pedigrees, 10 were novel (c.190T>G, c.199G>T, c.290G>C, c.322T>A, c.350_351insACAATAATTCCTA, c.391+5delG, c.416G>T, c.618_627delAGCTGAAACC, c.863delA, and c.1024_1027delACGA). These variants were not listed in ClinVar, HGMD, or the FIX Variant Database (http://www.factorix.org) as of February 2020. With our in‐house bioinformatics pipeline, the clinical significance each of these novel mutations was interpreted according to the ACMG criteria. The details are listed in Table 2. Of the 10 novel variants, four were predicted to be pathogenic (two nonsense mutations and two frameshift deletions), four were predicted to be likely pathogenic (all missense mutations), and two were predicted to be variants of unknown significance. As is described in Table 2, four missense mutations were predicted to be likely pathogenic according to ACMG guidelines, combined evidence to support the pathogenicity of these four missense mutations are PM1, PM2, PP2, PP3, PP4, PM1, and PM2 are moderate evidence of the pathogenicity. PP2, PP3, PP4 are supporting evidence.

**TABLE 2:** Novel mutations identified in 76 Chinese HB pedigrees

Exon	HGVS cDNA Name	HGVS Protein Name	Mutation Type	Significance*	Domain	CpG	Evidence
Exon2	c.190T>G	p.Cys64Gly	Missense	4	Gla	–	PM1, PM2, PP2, PP3, PP4
Exon2	c.199G>T	p.Glu67Ter	Nonsense	5	Gla	N	PVS1, PM1, PM2, PP3, PP4, PP5
Exon4	c.290G>C	p.Cys97Ser	Missense	4	EGF1	N	PM1, PM2, PP2, PP3, PP4
Exon4	c.322T>A	p.Cys108Ser	Missense	4	EGF1	N	PM1, PM2, PP2, PP3, PP4
Exon4	c.350_351insACAATAATTCCTA	p.C117_W118delinsTer	Nonsense	5	EGF1	N	PVS1, PM2, PP4
Intron 4	c.391+5delG	–	intronic	3	–	N	PM2, PP4
Exon5	c.416G>T	p.Gly139Val	Missense	4	EGF2	N	PM1, PM2, PP2, PP3, PP4
Exon6	c.618_627delAGCTGAAACC	p.Glu206fs	Deletion	5	Act‐Peptide	N	PVS1, PM2, PP4
Exon8	c.863delA	p.Glu288fs	Deletion	5	Serine Protease	–	PVS1, PM2, PP4
Exon8	c.1024_1027delACGA	p.Thr342fs	Deletion	5	Serine Protease	N	PVS1, PM2, PP4

* In the “significant” column, 5 refers to “pathogenic,” 4 refers to “likely pathogenic,” 3 refers to “variant of unknown significance.”

### 3.3 Families with double mutations

In the 76 HB pedigrees we analyzed, two pedigrees carried additional *F8* gene variants. *F9*:c.1231A>G(p.Ser411Gly) and *F8*:c.6490A>G(p.Ile2164Val) were both detected in one carrier and two patients from HB309. In the *F9* gene, c.1231A>G was the causative mutation in three HB cases in previous reports (Johnsen et al., 2017). The other mutation in the *F8* gene, c.6490A>G, was classified as likely pathogenic and is a novel mutation to our knowledge. Both of the variants were predicted to be “likely pathogenic” and validated by Sanger sequencing.

One patient from HB25 had a hemizygous *F9*:1294G>A(p.Gly432Ser) mutation and an *F8*:c.3169G>A(p.Ile2164Val) mutation. In the *F9* gene, c.1294G>A(p.Gly432Ser) was classified as likely pathogenic and has been recorded in HB patients from India, South Korea, Germany, and the United States (Johnsen et al., 2017; Kwon, Yoo, Kim, & Kim, 2008; Miller et al., 2012; Wulff, Schröder, Wehnert, & Herrmann, 1995).

### 3.4 Mosaic mutation

**FIGURE 2:**
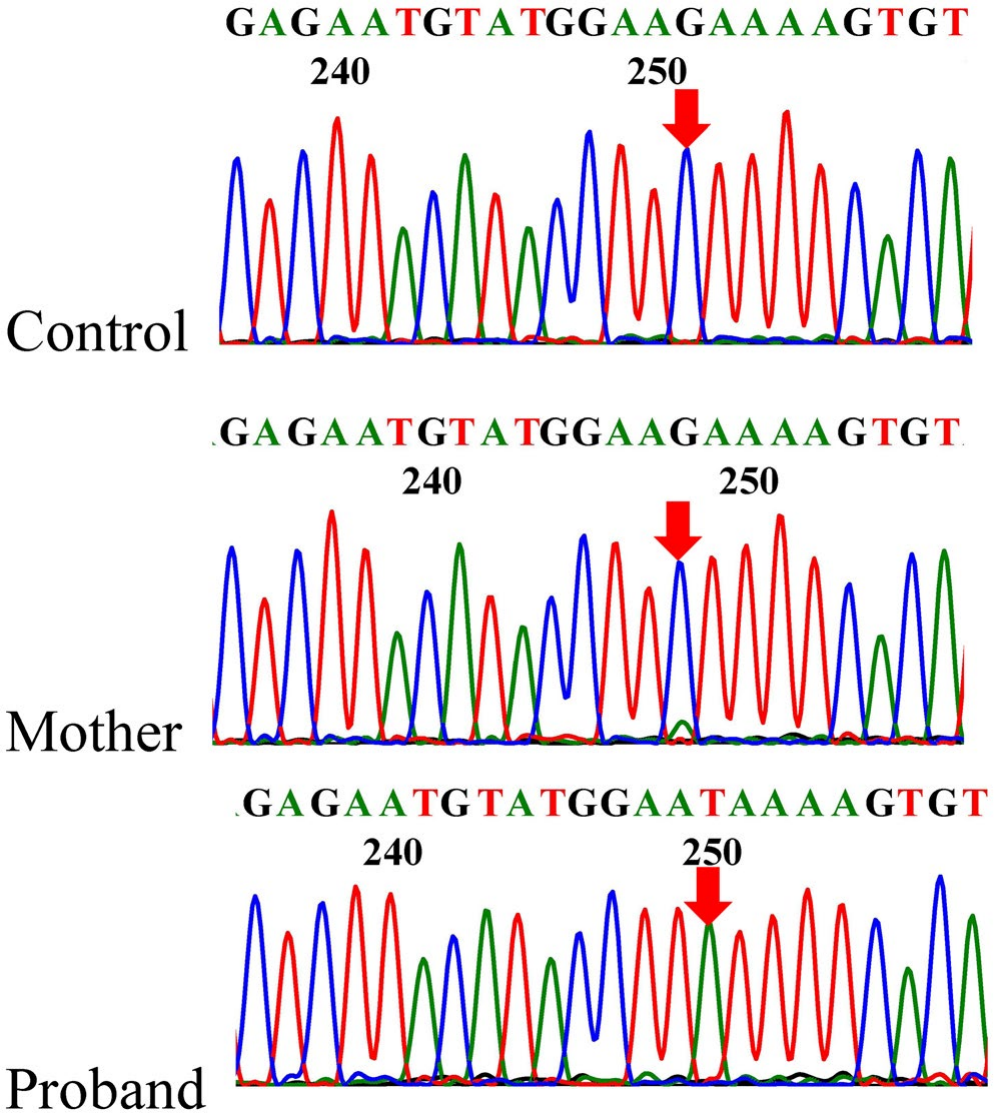
Sanger sequencing results for the *F9* gene. The proband in this pedigree was hemizygous for c.199G>T(p.Glu67Ter). The mother had a minor mutant T peak at the same location. The variants are indicated with red arrows

Of the 76 pedigrees analyzed, the mosaic mutation was discovered in one pedigree. The mutation c.199G>T(p.Glu67Ter) was initially detected only in patient of this pedigree. Subsequently, the proband's mother's BAM file was checked manually using IGV. The read depth at the site of this variant was 1730×, which showed that the allele T/G frequency was 9.36% (162/1730). The Sanger sequencing results are shown in Figure 2. Results showed that the mother carried a mosaic mutation.

## 4 DISCUSSION

With a detection rate of 94.74%, NGS was performed to analyze 76 unrelated Chinese HB pedigrees and 59 different variants were identified. Similar to previous reports, these mutations were distributed over the entire length of the *F9* gene.

Consistent with reported rates of 65%–70% (Goodeve, 2015), missense mutations accounted for 64.41% of the mutations identified in our study, mainly in serine protease domains (16/38, 42.11%). Of the 38 missense mutations we identified, 36 were categorized as likely pathogenic. The significance of the remaining two missense mutations (c.26C>G and c.59T>C) in the signal peptide is unknown. However, c.59T>C (p.Leu20Ser) has been reported in severe HB in China and Italy (Belvini et al., 2005; Liu et al., 2000).

Of three intronic mutations, two were splicing mutations (c.88+1G>T and c.391+2T>C) that could lead to the production of a truncated FIX protein. Although the predicted results showed the remaining mutation (c.391+5delG) could not create a new splice site, it may influence the gene expression effect in other ways. Besides, it was the only *F9* gene mutation detected in the proband, his mother, and a sibling. At the same location, c.391+5G>A was reported to be a splicing mutation (Ketterling et al., 1999). Interestingly, the FIX:C of the sibling was 38%, presenting as mild HB, consistent with reports that in rare symptomatic cases, female carriers were mildly symptomatic (Gangodkar et al., 2018).

It has been reported that 1.3%–7.8% of HB cases had more than one mutation (Goodeve, 2015). In our study, two of 76 HB pedigrees carried double candidate mutations (briefly mentioned in Li et al., 2020). One patient from pedigree HB25 carried the mutations c.3169G>A(p.Ile2164Val) in the *F8* gene and c.1294G>A(p.Gly432Ser) in the *F9* gene. Both were missense mutations. With frequencies of 0.00799 and 0.000198 in East and South Asians, respectively, c.3169G>A(p.Ile2164Val) in the *F8* gene seems more likely to be pathogenic (Lyu et al., 2016). In the EAHAD F9 mutation database, c.1294G>A(p.Gly432Ser) in the *F9* gene has been identified in patients at different centers (Johnsen et al., 2017; Kwon et al., 2008; Miller et al., 2012; Wulff et al., 1995). It remains unclear whether the two mutations work together to affect clinical severity. The detection of a second mutation is as important as the first in genetic diagnosis.

We discovered one sporadic case. The mother of the sporadic case had a mosaic c.199G>T(p.Glu67Ter) mutation in the *F9* gene. Minor peaks at mutation sites can easily be mistaken for the background peaks of such mosaic mutations, causing the mosaic mutation to be overlooked. However, genetic mosaicism may be not a rare event in sporadic hemophilia (Kasper & Buzin, 2009; Lannoy & Hermans, 2020; Lu et al., 2018). In a report that analyzed 804 hemophilia pedigrees, sporadic cases accounted for 30%–43% of all HB cases (Kasper & Lin, 2007). Thus, more consideration should be given to the prenatal diagnosis of sporadic hemophilia. Family based deep sequencing may help to discover mosaic mutations.

We were unable to find potentially causative mutations in four HB pedigrees, perhaps because the mutations that cause HB to locate in regions that our NGS panel did not cover (Johnsen et al., 2017). If the entire *F9* gene were examined, mutations might have been found in these four HB pedigrees. Deep intronic mutations that cause hemophilia A have been reported (Bach, Wolf, Oldenburg, Muller, & Rost, 2017; Pezeshkpoor et al., 2013). The role of microRNA in fine‐tuning *F8* gene regulation has also been emphasized (Jankowska et al., 2020). Thus, the entire *F9* gene sequence of these four HB pedigrees will be further determined in our study.

The major limitation of our study was that the residual plasma FIX activity of each pedigree was not complete enough to establish the genotype‐phenotype correlation. In addition, target regions were limited to find out potentially causative mutations in four HB pedigrees.

## 5 CONCLUSION

This study identified 59 different causative mutations in 76 Chinese pedigrees with HB. Ten novel mutations were reported: c.190T>G, c.199G>T, c.290G>C, c.322T>A, c.350_351insACAATAATTCCTA, c.391+5delG, c.416G>T, c.618_627delAGCTGAAACC, c.863delA, and c.1024_1027delACGA. This enriches the spectrum of *F9* mutations and provides further insight into the pathogenesis of HB in the Chinese population.

## CONFLICT OF INTEREST

The authors stated that they had no interests which might be perceived as posing a conflict or bias.

## AUTHORS' CONTRIBUTIONS

Xuexi Yang, Ming Li, and Qiang Li conceived and designed the study. Juanjuan Chen, Dongmei Fan, Xu Yang, and Yufeng Xiong performed the experiments. Wangjie Jin, Yihong Li, and Fenxia Li collected the clinical data and samples. Sheng Lin and Kun Li analyzed the sequencing data. Limin Huang and Liyan Li wrote the initial draft of the manuscript, Xuexi Yang, Ming Li, and Qiang Li revised the manuscript. All authors read and approved the manuscript.

## Supporting information

Table S1
