# Supplementary material for: Molecular analysis of 76 Chinese hemophilia B pedigrees and the identification of 10 novel mutations
Source: Mol Genet Genomic Med. 2020 Sep 1;8(11):e1482. doi: 10.1002/mgg3.1482 (PMC7667291; doi:10.1002/mgg3.1482)
Supplement: Supplementary file 1 — Table S1 [file MGG3-8-e1482-s001.docx]

**Supplementary TABLE 1** Basic information of 285 subjects from 76 unrelated HB

Chinese pedigrees.

| **Subject Numerber** | **Sample Name** | **Gender** | **Age**  **(years old)** | **Carrier State** |
| --- | --- | --- | --- | --- |
| 1 | HB5-1-1 | Female | NA | Normal |
| 2 | HB5-1-2 | Female | NA | Normal |
| 3 | HB5-1-3 | Female | NA | Normal |
| 4 | HB5-2-1 | Female | NA | Normal |
| 5 | HB5-2-2 | Male | 5 | Patient |
| 6 | HB5-2-3 | Female | 26 | Carrier |
| 7 | HB5-2-3b | Female | 27 | Normal |
| 8 | HB5-2-3d | Female | NA | Normal |
| 9 | HB5-2-4 | Female | NA | Carrier |
| 10 | HB5-2-5 | Female | NA | Normal |
| 11 | HB5-2-6 | Female | NA | Normal |
| 12 | HB5-2-7 | Male | NA | Normal |
| 13 | HB10-1-1 | Female | NA | Carrier |
| 14 | HB10-2-1 | Male | NA | Patient |
| 15 | HB10-2-2 | Female | NA | Normal |
| 16 | HB10-2-3 | Female | 27 | Carrier |
| 17 | HB10-2-4 | Female | NA | Normal |
| 18 | HB16-2-1 | Female | NA | Normal |
| 19 | HB16-2-2 | Female | 29 | Normal |
| 20 | HB16-3-1 | Male | NA | Patient |
| 21 | HB24-1-1 | Female | 21 | Carrier |
| 22 | HB24-1-2 | Female | NA | Carrier |
| 23 | HB24-2-1 | Female | NA | Carrier |
| 24 | HB24-2-2 | Male | NA | Patient |
| 25 | HB25-1-1 | Female | NA | Normal |
| 26 | HB25-1-2 | Male | 4 | Patient |
| 27 | HB26-1-1 | Female | 27 | Normal |
| 28 | HB26-1-2 | Male | 25 | Patient |
| 29 | HB43b | Male | 3 | Patient |
| 30 | HB60-1-1 | Female | 29 | Carrier |
| 31 | HB60-1-2 | Female | NA | Carrier |
| 32 | HB60-1-3 | Female | NA | Normal |
| 33 | HB60-1-4 | Female | NA | Carrier |
| 34 | HB60-1-6b | Male | NA | Normal |
| 35 | HB60-2-1 | Male | 4 | Patient |
| 36 | HB73-2-1 | Female | NA | Normal |
| 37 | HB73-2-2 | Female | 35 | Carrier |
| 38 | HB73-2-3 | Female | NA | Carrier |
| 39 | HB73-3-1 | Female | NA | Normal |
| 40 | HB73-3-2 | Female | 31 | Carrier |
| 41 | HB73-3-3 | Male | 7 | Patient |
| 42 | HB73-3-4 | Female | NA | Carrier |
| 43 | HB76-1-1 | Male | 27 | Normal |
| 44 | HB76-1-2 | Female | 24 | Carrier |
| 45 | HB76-2-1 | Male | 11 month | Patient |
| 46 | HB81-1-1 | Male | NA | Normal |
| 47 | HB81-1-2 | Female | NA | Carrier |
| 48 | HB81-2-1 | Female | NA | Normal |
| 49 | HB81-2-2 | Female | 30 | Normal |
| 50 | HB81-2-3 | Male | NA | Patient |
| 51 | HB81-3-1 | Female | NA | Normal |
| 52 | HB81-3-2 | Female | NA | Normal |
| 53 | HB94-1-1 | Female | 29 | Carrier |
| 54 | HB94-2-1 | Male | 3 | Patient |
| 55 | HB98-1-1 | Female | 27 | Carrier |
| 56 | HB98-2-1 | Male | 2 | Patient |
| 57 | HB100-1-1 | Female | NA | Carrier |
| 58 | HB100-1-2 | Female | NA | Carrier |
| 59 | HB100-1-3 | Female | 25 | Carrier |
| 60 | HB100-2-1 | Female | NA | Carrier |
| 61 | HB100-2-2 | Male | 4 | Patient |
| 62 | HB100-2-3 | Female | NA | Carrier |
| 63 | HB100-3-1 | Female | NA | Carrier |
| 64 | HB105-2-1 | Female | NA | Carrier |
| 65 | HB105-2-2a | Female | 26 | Normal |
| 66 | HB105-2-3 | Female | NA | Carrier |
| 67 | HB105-3-1 | Male | NA | Patient |
| 68 | HB106-1-1 | Male | 7 | Patient |
| 69 | HB106-2-1 | Female | 32 | Carrier |
| 70 | HB106C | Female | NA | Normal |
| 71 | HB130-1-1 | Female | NA | Normal |
| 72 | HB130-2-1 | Female | 28 | Carrier |
| 73 | HB130-2-2 | Female | NA | Normal |
| 74 | HB130-3-1 | Male | NA | Patient |
| 75 | HB131-1-1 | Male | NA | Normal |
| 76 | HB131-1-2 | Female | 23 | Carrier |
| 77 | HB131-2-1 | Male | NA | Patient |
| 78 | HB131-2-2 | Male | NA | Normal |
| 79 | HB131-2-3 | Female | NA | Carrier |
| 80 | HB133-1-1 | Male | NA | Normal |
| 81 | HB133-1-2 | Female | NA | Carrier |
| 82 | HB133-2-1 | Male | NA | Normal |
| 83 | HB133-2-2 | Female | NA | Carrier |
| 84 | HB133-2-3 | Male | NA | Normal |
| 85 | HB133-2-4 | Male | NA | Patient |
| 86 | HB133-2-5 | Female | NA | Normal |
| 87 | HB133-2-6 | Female | NA | Normal |
| 88 | HB133-3-1 | Female | 25 | Normal |
| 89 | HB133-3-2 | Female | NA | Carrier |
| 90 | HB133-3-3 | Male | NA | Patient |
| 91 | HB133-3-4 | Female | NA | Normal |
| 92 | HB133-3-5 | Female | NA | Normal |
| 93 | HB133-3-6 | Female | NA | Normal |
| 94 | HB137-1-1 | Female | 30 | Carrier |
| 95 | HB137-1-2 | Male | NA | Normal |
| 96 | HB137-2-1 | Male | NA | Patient |
| 97 | HB145-1-1 | Female | NA | Carrier |
| 98 | HB145-2-1 | Male | NA | Normal |
| 99 | HB145-2-2 | Female | 27 | Carrier |
| 100 | HB145-2-3 | Male | 26 | Patient |
| 101 | HB145-3-1 | Male | NA | Patient |
| 102 | HB146-1-1 | Female | NA | Normal |
| 103 | HB146-2-1 | Female | NA | Normal |
| 104 | HB146-2-2 | Female | NA | Normal |
| 105 | HB146-2-3 | Female | 23 | Carrier |
| 106 | HB146-2-4 | Female | NA | Normal |
| 107 | HB146-2-5 | Male | NA | Normal |
| 108 | HB146-3-1 | Female | NA | Normal |
| 109 | HB146-3-2 | Male | NA | Normal |
| 110 | HB146-3-3 | Male | NA | Patient |
| 111 | HB146-3-4 | Female | NA | Normal |
| 112 | HB156-1-1 | Female | 25 | Carrier |
| 113 | HB156-2-1 | Male | NA | Patient |
| 114 | HB175-1-1 | Female | 29 | Carrier |
| 115 | HB175-1-2 | Male | NA | Normal |
| 116 | HB175-2-1 | Male | NA | Patient |
| 117 | HB187-1-1 | Female | NA | Normal |
| 118 | HB187-2-1 | Female | 33 | Normal |
| 119 | HB187-3-1 | Male | NA | Patient |
| 120 | HB199-1-1 | Female | 29 | Carrier |
| 121 | HB199-2-1 | Female | NA | Normal |
| 122 | HB201-1-1 | Male | NA | Normal |
| 123 | HB201-1-2 | Female | 23 | Carrier |
| 124 | HB201-2-1 | Female | NA | Normal |
| 125 | HB202-1-1 | Female | 30 | Carrier |
| 126 | HB202-2-1 | Female | NA | Normal |
| 127 | HB221-1-1 | Female | 29 | Carrier |
| 128 | HB221-2-1 | Female | NA | Normal |
| 129 | HB221-2-2 | Male | NA | Patient |
| 130 | HB227-2-1 | Female | NA | Normal |
| 131 | HB227-2-2 | Male | NA | Patient |
| 132 | HB255-1-1 | Female | 28 | Carrier |
| 133 | HB255-2-1 | Male | 3 | Patient |
| 134 | HB257-1-1 | Female | NA | Carrier |
| 135 | HB257-2-1 | Female | 30 | Carrier |
| 136 | HB257-2-2 | Female | NA | Normal |
| 137 | HB257-3-1 | Male | 9 | Patient |
| 138 | HB257-3-2 | Female | 6 | Normal |
| 139 | HB257-3-3 | Female | NA | Normal |
| 140 | HB258-1-1 | Female | NA | Carrier |
| 141 | HB258-2-1 | Female | 31 | Carrier |
| 142 | HB258-3-1 | Male | 6 | Patient |
| 143 | HB337-2-1 | Female | 27 | Carrier |
| 144 | HB337-2-2 | Male | 25 | Patient |
| 145 | HB433a | Female | 28 | Carrier |
| 146 | HB433b | Male | 31 | Normal |
| 147 | HB439-2-1 | Female | 31 | Carrier |
| 148 | HB439-3-1 | Male | 8 | Patient |
| 149 | HB486-2-1 | Female | NA | Carrier |
| 150 | HB486-3-1 | Male | 4 | Patient |
| 151 | HB486b | Male | NA | Normal |
| 152 | HB212-1-1 | Female | 28 | Carrier |
| 153 | HB212-2-1 | Male | NA | Patient |
| 154 | HB233-1-1 | Female | 26 | Carrier |
| 155 | HB233-2-1 | Male | NA | Patient |
| 156 | HB233-2-2 | Male | NA | Patient |
| 157 | HB233-2-3 | Male | NA | Patient |
| 158 | HB235-1-1 | Female | 31 | Carrier |
| 159 | HB235-2-1 | Male | 5 | Patient |
| 160 | HB239-1-1 | Female | NA | Carrier |
| 161 | HB239-2-1 | Male | 3 | Patient |
| 162 | HB253-2-1 | Female | 24 | Carrier |
| 163 | HB254-1-1 | Female | NA | Carrier |
| 164 | HB254-2-1 | Female | 33 | Normal |
| 165 | HB254-2-2 | Female | NA | Carrier |
| 166 | HB254-3-1 | Female | NA | Normal |
| 167 | HB254-3-2 | Male | NA | Patient |
| 168 | HB265-1-1 | Female | NA | Carrier |
| 169 | HB265-1-2 | Female | NA | Normal |
| 170 | HB265-1-3 | Male | NA | Normal |
| 171 | HB265-2-1 | Male | NA | Patient |
| 172 | HB265-2-2 | Female | 26 | Normal |
| 173 | HB287-1-1 | Female | NA | Carrier |
| 174 | HB287-2-1 | Female | NA | Carrier |
| 175 | HB287-2-2 | Female | 29 | Normal |
| 176 | HB287-3-1 | Male | NA | Patient |
| 177 | HB292-1-1 | Male | 36 | Patient |
| 178 | HB292-1-2 | Male | NA | Patient |
| 179 | HB292-2-1 | Female | NA | Carrier |
| 180 | HB298-1-1 | Female | NA | Normal |
| 181 | HB298-2-1 | Female | NA | Carrier |
| 182 | HB298-3-1 | Male | 3 | Patient |
| 183 | HB299-1-1 | Female | NA | Normal |
| 184 | HB299-2-1 | Female | NA | Carrier |
| 185 | HB299-2-2 | Female | NA | Normal |
| 186 | HB299-2-3 | Female | NA | Normal |
| 187 | HB299-2-4 | Female | 30 | Normal |
| 188 | HB299-2-5 | Female | NA | Normal |
| 189 | HB299-3-1 | Male | NA | Patient |
| 190 | HB299-3-2 | Female | NA | Normal |
| 191 | HB304-1-1 | Female | 28 | Normal |
| 192 | HB304-1-1b | Male | NA | Normal |
| 193 | HB304-2-1 | Male | NA | Patient |
| 194 | HB308-1-1 | Male | NA | Patient |
| 195 | HB308-2-1 | Female | 30 | Normal |
| 196 | HB309-2-1 | Female | NA | Carrier |
| 197 | HB309-3-1 | Male | 7 month | Patient |
| 198 | HB309-3-2 | Male | NA | Patient |
| 199 | HB309-3-3 | Female | NA | Normal |
| 200 | HB309-3-4 | Female | NA | Normal |
| 201 | HB334-1-1 | Female | 28 | Carrier |
| 202 | HB334-1-1b | Male | NA | Normal |
| 203 | HB348-1-1 | Male | 52 | Patient |
| 204 | HB348-2-1 | Female | NA | Carrier |
| 205 | HB348-2-2 | Female | NA | Carrier |
| 206 | HB355-2-1 | Female | 28 | Carrier |
| 207 | HB355-2-2 | Female | NA | Normal |
| 208 | HB355-3-1 | Male | NA | Patient |
| 209 | HB356-1-1 | Female | NA | Carrier |
| 210 | HB356-2-1 | Male | NA | Patient |
| 211 | HB356-2-2 | Female | 41 | Normal |
| 212 | HB358-2-1 | Female | 24 | Carrier |
| 213 | HB358-2-2 | Male | NA | Patient |
| 214 | HB364-2-1 | Female | 38 | Carrier |
| 215 | HB364-3-1 | Female | NA | Normal |
| 216 | HB364-3-2 | Female | NA | Normal |
| 217 | HB376-1-1 | Female | NA | Carrier |
| 218 | HB376-2-1 | Male | NA | Patient |
| 219 | HB376-2-2 | Female | 31 | Carrier |
| 220 | HB376-2-2b | Male | NA | Normal |
| 221 | HB376-2-3 | Male | NA | Normal |
| 222 | HB386-1-1 | Female | NA | Normal |
| 223 | HB386-1-2 | Female | NA | Carrier |
| 224 | HB386-1-3 | Female | NA | Normal |
| 225 | HB386-1-4 | Female | 28 | Normal |
| 226 | HB386-2-1 | Female | NA | Normal |
| 227 | HB386-2-2 | Male | NA | Patient |
| 228 | HB390-1-1 | Female | NA | Carrier |
| 229 | HB390-2-1 | Male | NA | Normal |
| 230 | HB390-2-2 | Female | 23 | Carrier |
| 231 | HB390-2-2b | Male | NA | Normal |
| 232 | HB390-2-3 | Female | NA | Normal |
| 233 | HB390-2-4 | Male | NA | Patient |
| 234 | HB390-3-1 | Male | NA | Patient |
| 235 | HB409-2-1 | Female | 29 | Carrier |
| 236 | HB409-3-1 | Male | NA | Patient |
| 237 | HB415-2-1 | Female | 35 | Carrier |
| 238 | HB415-2-2 | Male | NA | Normal |
| 239 | HB415-3-1 | Male | 6 | Patient |
| 240 | HB423-2-1 | Female | NA | Carrier |
| 241 | HB423-3-1 | Female | NA | Carrier |
| 242 | HB423-3-2 | Male | 5 | Patient |
| 243 | HB444-2-1 | Female | 26 | Normal |
| 244 | HB444-2-1b | Male | NA | Normal |
| 245 | HB444-2-2 | Female | NA | Carrier |
| 246 | HB444-2-3 | Male | NA | Patient |
| 247 | HB445-1-1 | Female | NA | Normal |
| 248 | HB445-2-1 | Female | NA | Normal |
| 249 | HB445-2-2 | Female | 24 | Carrier |
| 250 | HB445-3-1 | Male | 2 | Patient |
| 251 | HB450-2-1 | Female | 35 | Normal |
| 252 | HB450-3-1 | Male | NA | Patient |
| 253 | HB456-1-1 | Female | NA | Normal |
| 254 | HB456-2-1 | Female | 29 | Carrier |
| 255 | HB456-2-2 | Female | NA | Normal |
| 256 | HB456-2-3 | Female | NA | Normal |
| 257 | HB456-2-4 | Female | NA | Normal |
| 258 | HB456-3-1 | Male | 3 | Patient |
| 259 | HB458-1-1 | Female | NA | Carrier |
| 260 | HB458-2-1 | Female | 23 | Carrier |
| 261 | HB458-2-2 | Male | NA | Patient |
| 262 | HB462-2-1 | Female | 34 | Carrier |
| 263 | HB465-2-1 | Male | NA | Patient |
| 264 | HB465-2-2 | Female | 32 | Carrier |
| 265 | HB472a | Female | 31 | Carrier |
| 266 | HB484-2-1 | Female | NA | Carrier |
| 267 | HB484-3-1 | Male | 6 | Patient |
| 268 | HB495-1-1 | Female | NA | Carrier |
| 269 | HB495-1-2 | Female | 28 | Carrier |
| 270 | HB495-1-3 | Female | NA | Carrier |
| 271 | HB495-1-4 | Female | NA | Carrier |
| 272 | HB495-2-1 | Female | NA | Normal |
| 273 | HB495-2-2 | Male | 3 | Patient |
| 274 | HB495-2-3 | Female | NA | Carrier |
| 275 | HB495-2-4 | Female | NA | Normal |
| 276 | HB508-2-1 | Female | 26 | Carrier |
| 277 | HB508-3-1 | Male | 2 | Patient |
| 278 | HB514-2-1 | Female | 26 | Carrier |
| 279 | HB514-2-2 | Female | 27 | Carrier |
| 280 | HB514-3-1 | Male | 4 | Patient |
| 281 | HB514-3-2 | Female | 2 | Carrier |
| 282 | HB517-1-1 | Female | 58 | Carrier |
| 283 | HB517-2-1 | Female | 37 | Carrier |
| 284 | HB517-2-2 | Male | NA | Normal |
| 285 | HB517-3-1 | Male | NA | Patient |

NA, not available.
